# An autopsied FTDP-17 case with MAPT IVS 10 + 14C > T mutation presenting with frontotemporal dementia

**DOI:** 10.1016/j.ensci.2021.100363

**Published:** 2021-07-28

**Authors:** Ryohei Watanabe, Ito Kawakami, Takeshi Ikeuchi, Shigeo Murayama, Tetsuaki Arai, Haruhiko Akiyama, Mitsumoto Onaya, Masato Hasegawa

**Affiliations:** a Dementia Research Project, Tokyo Metropolitan Institute of Medical Science, 2-1-6 Kamikitazawa, Setagaya, Tokyo, Japan; b Department of Psychiatry, University of Tsukuba, 1-1-1 Tennodai, Tsukuba, Ibaraki, Japan; c Department of Molecular Genetics, Brain Research Institute, Niigata University, 1–757 Asahimachi, Niigata, Niigata, Japan; d Brain Bank for Aging Research, Department of Neurology, Tokyo Metropolitan Institute of Gerontology, 35-2 Sakaecho, Itabashi, Tokyo, Japan; e Department of Clinical Research, Yokohama Brain and Spine Center, 1-2-1 Takigashira, Isogo, Yokohama, Kanagawa, Japan; f Department of Psychiatry, National Hospital Organization Shimofusa Psychiatric Medical Center, 578 Hetacho, Midori, Chiba, Chiba, Japan

**Keywords:** FTDP-17, MAPT, Mutation, FTLD, FTD, Neuropathology

## Abstract

• We report the immunohistochemical and biochemical features of an FTDP-17 case with MAPT IVS 10 + 14C > T mutation.
• Postmortem examination of the patient with bvFTD revealed diffuse neuronal and glial 4-repeat tau pathology similar to CBD.
• The structure of tau filaments associated with MAPT IVS 10 + 14C > T mutation was characterized by electron microscopy.

Dear Editor,

Frontotemporal dementia and parkinsonism linked to chromosome 17 (FTDP-17) due to *MAPT* mutation is a heterogeneous genetic neurodegenerative disorder associated with familial frontotemporal dementia (FTD) and/or parkinsonism [[1], [2], [3]]. Many mutational loci have been defined in the *MAPT* gene on chromosome 17, which encodes tau protein [3], and mutations of *MAPT* can cause brain pathology resembling frontotemporal lobar degeneration with tau inclusions (FTLD-tau) [4]. However, there are few reports on the neuropathology of some mutations, and the pathogenesis has not been fully elucidated. Here, we describe the pathological and biochemical features of an FTDP-17 case harboring the very rare *MAPT* intervening sequence (IVS) 10 + 14C > T mutation.

## 1 Case presentation

**Fig. 1:**
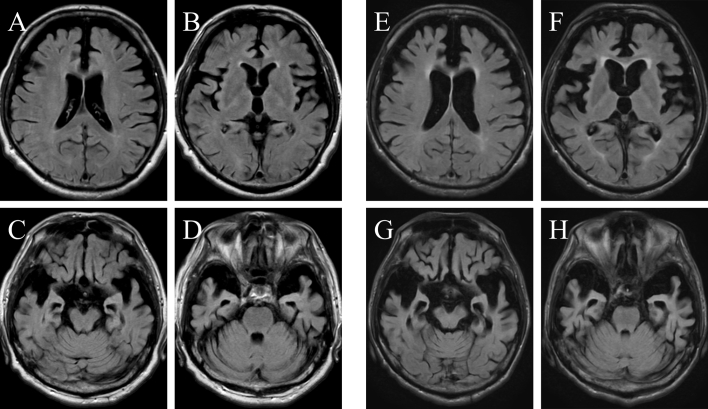
Horizontal T1-weighted brain MR images at age 53 (A-D) and age 58 (*E*-H), 6 and 11 years after onset, respectively. The images show progression of the marked atrophy of the bilateral frontal lobes (A, B, E, F), and the temporal lobes including hippocampus (C, D, G, H). The involvement of white matter is mild and brainstem regions, such as the pons and midbrain, are relatively preserved.

A Japanese male in his late 40s, with no obvious neuropsychiatric family history, presented stereotyped behavior, disinhibition, and loss of sympathy, which gradually became more apparent. Hyperorality, dietary changes, repetitive speech, hoarding, and apathy followed within a few years. Donepezil was not effective, and at age 51, he was admitted to our hospital. Neurological examination and blood test findings were normal. The Cognistat test showed normal orientation but impaired memory, language, and reasoning function, and the Frontal Assessment Battery (FAB) score was 14/18, indicating slight frontal lobe dysfunction. Brain MR imaging revealed marked atrophy of the bilateral hippocampus, amygdala, and frontal and anterior temporal lobes (Fig. 1). The patient was diagnosed as probable behavioral variant FTD (bvFTD) [5]. The behavioral symptoms improved slightly on treatment with carbamazepine and valproic acid, but the cognitive dysfunction progressed. The patient's spontaneous activity gradually decreased, followed by muscle wasting, although other motor symptoms including parkinsonism were not apparent in the advanced stages. The patient eventually became bedridden and died of aspiration pneumonia at age 58.

**Fig. 2:**
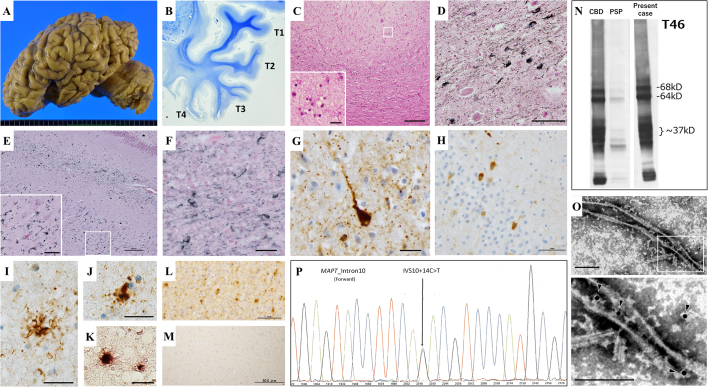
Neuropathological, biochemical, and genetic findings. A: Severe macroscopic atrophy of the left frontal lobe and temporal pole. B: Klüver-Barrera staining of the temporal lobe shows severe atrophy in the lower parts (T3-4). C: Hematoxylin-eosin staining of the inferior temporal cortex shows marked neuronal loss and gliosis. The inserted image shows increased astrocytes. D–F: Gallyas-Braak staining shows diffuse dystrophic neurites and coiled bodies in amygdala (D) and hippocampal CA1 (E with a magnified image) and threads and coiled bodies in the white matter adjacent to the middle temporal cortex (F). G–M: Neuronal and glial inclusions were positive for phosphorylated tau. G: PS422 staining of the middle temporal cortex showing a pretangle and diffuse dot-like dystrophic neurites. H: PHF1 staining shows some pretangles and diffuse dystrophic neurites in the dentate gyrus. I: A tuft-shaped astrocyte in the inferior temporal cortex detected by AT8 antibody. *J*, K: Globular inclusions in the proximal processes of astrocyte in the inferior temporal cortex detected by PS422 (*J*) and AT180 (K) antibodies. L, M: Tau deposits were detected by anti-4R (L) but not by RD3 (M) antibody in the white matter of the middle temporal lobe. N: T46 antibody immunoblot of the brain extract showing a strong 37 kDa band similar to CBD. A PSP brain sample was also analyzed for comparison. O: Immunoelectron microscopy of the brain extract shows ribbon-like twisted tau filaments labelled with AT8 antibody (indicated with arrowheads in the enlarged image). P: Sanger sequence showing the *MAPT* IVS 10 + 14C > T mutation. Scale bar in each image represents: 100 μm (C, E), 50 μm (D, H, L, M), 20 μm (insets of C and E, F, G, I–K), or 100 nm (O).

Postmortem examination showed macroscopically severe brain atrophy in the bilateral cerebral frontotemporal lobes (Fig. 2A). The temporal lobe showed severer atrophy in the lower parts (T3-4) than in the upper parts (T1-2) (Fig. 2B), and atrophy of the caudate nuclei, amygdala, and hippocampus was also apparent. The brainstem was preserved, and the substantia nigra and locus coeruleus showed mildly reduced pigmentation. Histologically, neuronal loss and gliosis were the most pronounced in the middle and inferior temporal cortices (Fig. 2C), entorhinal cortex, hippocampus, and striatum. In the locus coeruleus and substantia nigra, some free melanin was found, but neuronal loss was not apparent. Degeneration of precentral gyrus and pyramidal tract was not evident. Gallyas-Braak (GB) staining revealed diffuse neuronal and glial staining in the cerebral neocortex, limbic regions, subcortical nuclei, and white matter (Fig. 2D–F). Immunostaining revealed phosphorylated 4-repeat tau inclusions (Fig. 2G–M), as summarized in Table 1. The major neuronal tau inclusions were GB-positive dots and thread-like dystrophic neurites. Pretangles were also seen but were not very frequent (Fig. 2E, G, H). Glial deposits were mostly GB-positive oligodendroglial inclusions and coiled bodies (Fig. 2F). Astrocytic inclusions, observed as tuft-shaped (Fig. 2I) or globular inclusions (Fig. 2J, K), were considerably fewer and were GB-negative. The accumulation of tau deposits was less in the brainstem and cerebellum. The pathological hallmarks of CBD, such as prominent ballooned neurons and astrocytic plaques, were absent. Argyrophilic grains were moderately seen in amygdala and related limbic cortexes. Anti-phosphorylated synuclein immunostaining detected only a few Lewy neurites in the hippocampus and temporal cortex, and methenamine‑silver staining and anti-phosphorylated TDP-43 immunostaining were consistently negative. Immunoblotting of the sarkosyl-insoluble fraction from the temporal lobes revealed a 4-repeat tau banding pattern with predominant 37 kDa fragments (Fig. 2N). Electron micrographs of sarkosyl-insoluble fraction from the temporal lobes with AT8 antibody labeling revealed ribbon-like filamentous structures with a regular twist at approximately every 200 nm (Fig. 2O). The DNA sequence analysis revealed the presence of *MAPT* IVS 10 + 14C > T heterozygous mutation in the frontal cortex (Fig. 2P).

**Table 1:** Neurodegeneration and tau-positive deposits in each brain region are classified as severe (++), moderate (+), mild (+/−), or not apparent (−).

	NEURONAL loss/gliosis	Neuronal tau inclusions	Glial tau inclusions
Cortical areas			
Frontal	+	+	+
Motor	+	+/−	+
Parietal	+	+/−	+
Temporal	++	++	++
Entorhinal	++	++	++
Subiculum	+	++	++
Hippocampal CA	++	++	++
Cingulate	+	+	+
Occipital	+/−	NA	NA

Subcortical areas			
White matter	++	−	++
Nucleus Accumbens	++	NA	NA
Amygdala	+	+	+
Striatum	++	++	++
Globus pallidus	+	++	++
Thalamus	−	+	+
Subthalamic nucleus	++	++	+

Midbrain			
Red nucleus	−	+	+
Substantia nigra	+/−	+	+

Pons			
Locus ceruleus	+	+	+
Pontine nuclei	+/−	+	+

Medulla oblongata			
Hypoglossal nucleus	−	−	−
Inferior olivary nucleus	+/−	−	−

Cerebellum			
Purkinje cell	+	+	−
Granule cell	−	+	−
Dentate nucleus	+	+	+

## 2 Discussion

FTDP-17 with *MAPT* mutation is extremely rare. There have been only two reports of neuropathologically examined IVS 10 + 14C > T mutation, with seven autopsied patients in total (Table 2), and detailed immunohistochemical and biochemical analyses were performed only in one case [1,6].

**Table 2:** Summary of cases of FTDP-17 with *MAPT* IVS 10 + 14C > T mutation.

	Lynch T (1994) [1]	Omoto M (2012) [6]	Present case
Age of death	58 on average	54	58
Gender	Males and females	Female	Male
Number of autopsied cases	6	1	1
Family linkage	13 members in an Irish-American family	2 Japanese siblings	NA
Age at onset	45 on average	44	Late 40s
Disease duration	13 years on average	10 years	11 years
Psychiatric symptoms	Changes in personality, amnesia, poor construction with preservation of orientation, speech, and calculation	Apathy followed by memory loss and disorientation	Changes in personality, disinhibition, apathy, dietary changes, and stereotypy followed by memory loss and disorientation
Neurological findings	Rigidity, bradykinesia,postural instability, fasculation	Mask-like face, hypophonic voice, bradykinesia,right-sided rigidity, hyperactive deep tendon reflex	Dysphagia
Clinical diagnosis	disinhibition-dementia-parkinsonism-amyotrophy complex	FTDP-17	behavioral variant FTD
Major affected brain regions	Frontal and temporal cortex, amygdalae, and substantia nigra	Globus pallidus, cerebellum, subthalamic nucleus, substantia nigra, and locus coeruleus	Middle and inferior temporal cortex, entorhinal cortex, hippocampus, and striatum.
Tau isoform	NA	4-repeat	4-repeat
Biochemical analysis	NA	PSP pattern	CBD pattern
		(predominant 33 kDa bands)	(predominant 37 kDa bands)
Tau filament subtype	NA	NA	Ribbon-like twisted filament
Mutational site	*MAPT* IVS 10 + 14C > T		

Cases with this mutation clinically presented both psychiatric and neurological symptoms in various proportions. Our case showed moderate involvement of the prefrontal and anterior cingulate cortices, which may account for the behavioral symptoms [7]. Atrophy of the medial prefrontal cortex along the dilated longitudinal fissure resembled that in a previously reported bvFTD case harboring *MAPT* IVS 10 + 3 mutation [3]. Our case also showed severe pathology in the limbic regions, which, in general, are associated with behavioral responses. A recent study indicated that FTDP-17 may exhibit greater volume loss of the amygdala than the other FTLD subtypes [8]. Limbic-prefrontal dysfunction might have accounted for our case's behavioral symptoms. We found that neurological symptoms, such as parkinsonism, were not prominent and the substantia nigra was relatively preserved in comparison with previous cases [6]. Such variability in pathology might account for the heterogeneity of clinical phenotypes.

All reported IVS10 mutations [2,3,9] were associated with 4-repeat tauopathies such as corticobasal degeneration (CBD), progressive supranuclear palsy (PSP), and recently proposed globular glial tauopathy (GGT) [10]. In the present case, the biochemical findings correspond well with the CBD pattern, but the pathology was atypical for CBD. Immunohistochemistry showed common CBD pathological features, such as abundant pretangles, dystrophic neurites, and glial inclusions, but the pathological hallmarks of CBD such as prominent ballooned neurons and astrocytic plaques were absent, and the main pathology was focused on the cerebral cortex and hippocampus, with only mild neurodegeneration in the substantia nigra. Moreover, a small number of pathological structures exhibited PSP-like features. These results collectively suggest a heterogeneous and multiple tau pathology, but not typical CBD or PSP, and the IVS mutation might be related to this. In addition, we identified electron-microscopic tau filaments presumably caused by the IVS 10 + 14 mutation. The appearance of those ribbon-like filamentous structures was similar to that seen in adjacent IVS mutations [2]. Similarities of filamentous tau structure due to different IVS mutations might cause specific clinical and pathological phenotypes [3]. Further evidence is needed to clarify the genetic-pathological-clinical correlations in detail.

## Funding sources

This study was supported by funds from The 10.13039/100007428Naito Foundation (to IK), 10.13039/100009619Japan Agency for Medical Research and Development (AMED) under Grant Numbers JP21dk0207045 (to TI), JP18ek0109391 and JP18dm0207019 (to MH), and 10.13039/501100002241Japan Science and Technology Agency (JST) CREST Grant Number JPMJCR18H3 (to MH). The funding sources had no role in the design of this study.

## Ethics approval and consent

The patient's next to kin gave written consent for autopsy and postmortem analysis for research purposes. This study was approved by the ethics committee in the Tokyo Metropolitan Institute of Medical Science and was performed in accordance with the ethical standards laid down in the 1964 Declaration of Helsinki and its later amendments.

## Declaration of interest

None.

## References

[1] Lynch T., Sano M., Marder K.S., Bell K.L., Foster N.L. (1994 Oct). Clinical characteristics of a family with chromosome 17-linked disinhibition-dementia-parkinsonism-amyotrophy complex. Neurology.

[2] Goedert M., Spillantini M.G. (2000 Jul). Tau mutations in frontotemporal dementia FTDP-17 and their relevance for Alzheimer’s disease. Biochim. Biophys. Acta.

[3] Ghetti B., Oblak A.L., Boeve B.F., Johnson K.A., Dickerson B.C. (2015 Feb). Invited review: Frontotemporal dementia caused by microtubule-associated protein tau gene (MAPT) mutations: a chameleon for neuropathology and neuroimaging. Neuropathol. Appl. Neurobiol..

[4] Forrest S.L., Kril J.J., Stevens C.H., Kwok J.B., Hallupp M. (2018 Feb). Retiring the term FTDP-17 as MAPT mutations are genetic forms of sporadic frontotemporal tauopathies. Brain.

[5] Rascovsky K., Hodges J.R., Knopman D., Mendez M.F., Kramer J.H. (2011). Sensitivity of revised diagnostic criteria for the behavioural variant of frontotemporal dementia. Brain.

[6] Omoto M., Suzuki S., Ikeuchi T., Ishihara T., Kobayashi T. (2012 Mar). Autosomal dominant tauopathy with parkinsonism and central hypoventilation. Neurology.

[7] Firat R.B. (2019 Feb). Opening the “Black Box”: functions of the frontal lobes and their implications for sociology. Front. Sociol..

[8] Bocchetta M., Iglesias J.E., Cash D.M., Warren J.D., Rohrer J.D. (2019 Dec). Amygdala subnuclei are differentially affected in the different genetic and pathological forms of frontotemporal dementia. Alzheimers Dement..

[9] Umeda T., Yamashita T., Kimura T., Ohnishi K., Takuma H. (2013 Jul). Neurodegenerative disorder FTDP-17-related tau intron 10 +16C → T mutation increases tau exon 10 splicing and causes tauopathy in transgenic mice. Am. J. Pathol..

[10] Ahmed Z., Bigio E.H., Budka H., Dickson D.W., Ferrer I. (2013 Oct). Globular glial tauopathies (GGT): consensus recommendations. Acta Neuropathol..

